# Special Issue: Treatments for Fungal Infections

**DOI:** 10.3390/jof4040135

**Published:** 2018-12-12

**Authors:** Esther Segal, Daniel Elad

**Affiliations:** 1 Department of Clinical Microbiology and Immunology, Sackler School of Medicine, Tel Aviv University, Tel Aviv 69978, Israel; 2 Department of Veterinary Clinical Bacteriology and Mycology, The Kimron Veterinary Institute, Bet Dagan 50250, Israel

Antifungal therapy is complicated compared to antibacterial treatments by the fact that fungi and their hosts are both eukaryotic organisms, resulting in fewer targets for selective activity. Thus, the variety of early antimycotics was limited and aimed primarily at the topical treatment of superficial mycoses until the 1980s. The few drugs that could be used systemically for invasive fungal infections were often hampered by toxic effects. The subsequent increase in systemic mycoses, resulting mostly from pathological and iatrogenic states of immunosuppression, necessitated the development of new drugs for systemic administration having a broader spectrum, lower toxicity and better pharmacodynamic/pharmacokinetic characteristics. This led to the production of new and improved azoles and polyene formulations, as well as a new family of drugs—the echinocandins. Several antimycotic drugs currently in various phases of development have novel molecular targets and they are unlikely to be subject to cross resistance with existing drugs [1,2,3,4]. It is noteworthy that since immunosuppression is the predisposing factor, to a large part, for invasive fungal infections, the restoration of a functional immune system as soon as possible is of essence in achieving therapeutic success [5], keeping in mind the possible complications derived from the immune reconstitution inflammatory syndrome (IRIS).

In this Special Issue, the treatment of major invasive human mycoses is covered, including candidiasis, cryptococcosis, aspergillosis, mucormycosis, fusariosis, scedosporiasis and pneumocystis infection, as well as skin, hair and nail infections. Moreover, special attention was directed towards pediatric patients. Additional topics include susceptibility testing and resistance to antifungal drugs. Finally, the special aspects of the treatment of veterinary mycoses are reviewed.

An important topic in medical mycology in recent years is resistance to antimycotic drugs [6]. Resistance may be intrinsic, as is observed for *Aspergillus terreus* to amphotericin B [7] or as noted for *Candida krusei* to fluconazole [1]. In the latter case, mutation(s) making the microorganism resistant may be already present at the time of infection or evolve subsequently, especially during long periods of exposure to the drug, as is necessary in prophylactic treatments. 

*C. glabrata* is especially prone to develop resistance during therapy due to its ability to go through a slow-growing persistence phase, providing the necessary time to develop resistant mutants [8]. Low concentrations of echinocandins in abscesses caused by *C. glabrata* may be a further mechanism of resistance emergence [8]. Moreover, some fungal pathogens have variable susceptibility profiles [5]. Consequently, to choose the optimal antimycotic treatment, susceptibility testing is necessary [9]. 

The two main standards for antimycotic susceptibility testing are those of the American CLSI and European EUCAST. In their review, Sanguinetti et al. [6] present the similarities and differences between the two standards. Moreover, the pros and cons of the different methodologies (broth microdilution dilution, agar based, automated and analytical) are discussed, as are those of the clinical and epidemiological breakpoints. 

Several technical caveats in performing antifungal susceptibility tests are listed in the reviews in this Special Issue: susceptibility of *Candida* should not be tested with caspofungin as results are not uniform [1]; to reveal resistance of *A. fumigatus* both itraconazole and voriconazole should be tested, while results for posaconazole may be difficult to interpret due to the overlap of resistant and wild type values [9].

Molecular methods have been developed, but since they are directed towards the detection of specific resistance genes, strains with other mechanisms may be missed [9]. 

The importance of initiating antimycotic therapy against invasive fungal infections as early as possible is repeatedly stressed in the reviews of this Special Issue [1,4,7,10,11]. Consequently, considering the time required for isolation, identification and susceptibility testing, the initial therapy is often empiric. For some mycoses, biomarkers and risk scores have been formulated [1]. Prophylactic therapy is often used for high at-risk patients. However, unrecognized predisposing factors may preclude its timely start [12]. 

For the more common infections, accepted therapeutic approaches have been formulated: itraconazole or voriconazole for mycoses caused by fungi of the scedosporium/pseudallescheria/lomentospora complex [5]; amphotericin B and/or new trazoles for mucormycosis [4]; itraconazole or voriconazole for aspergillosis [10]; polyenes and fluconazole for cryptococcosis; and the latter as prophylaxis for patients with antigenemia below 1:160 [3]. 

*Pneumocystis* is a special case in several aspects: susceptibility testing is hindered by an inability to grow the organism on artificial media, and the lack of ergosterol in the organism’s cell membrane, which renders it less susceptible to polyenes and azoles, but susceptible to antibacterials such as sulfamethoxazole-trimethoprim [12].

Children/neonates present special challenges, such as the difficulty or impossibility of obtaining information directly from the patient and the need to adapt therapeutic protocols to the anatomy and physiology of this population [11]. Antifungal treatment in pediatric patients often involves prophylaxis in at-risk populations, such as neonates and immunosuppressed patients, or therapeutic intervention for treatment of active mycoses. Special pediatric protocols for antifungal therapy have been devised [11].

The most prevalent etiology of mycotic skin infections are dermatophytes, *Candida* spp. and *Malassezia* spp. Dermtophytoses may be treated topically or systemically, with azoles or allylamines, primarily terbibafine. Griseofulvin is preferred for the treatment of pediatric scalp infections in some countries. Since *Trichophyton* spp. are more susceptible to terbinafine and *Microsporum* spp. to griseofulvin, the identification of the etiological agent is of importance in choosing the optimal therapy. Nail dermatophytosis is characterized by requiring extended periods of treatment leading to low compliance and a high rate of recurrence due to incomplete microbiological cure. This has promoted efforts to develop new drugs with better nail penetration and less demanding application procedures. Skin and mucosal candidiasis may be treated with polyenes or azoles (topically) and triazoles (systemically). Azoles and allylamines are used also for the topical treatment of pityriasis versicolor, whereas itraconazole may be administered systemically. Shampoos are available for adjunct treatment of large affected skin areas [2].

In some cases, the antimycotic drug therapy must be complemented by other means, such as surgical source control in cases of mucormycosis or *Candida* abscesses [1,4], catheter removal [1] or application of laser or photodynamic processes for dermatophytosis [2]. Since *Pneumocystis* is transmissible between susceptible populations, adequate precautions must be taken [12].

To complete the picture of antimycotic therapy and in the spirit of One Health, aspects specific for the treatment of veterinary mycoses are presented. Since the treatment of animal dermatophytosis has been recently addressed [13], the focus of the present review concentrates on other fungal infections. Animal mycoses differ from human infections in the variety of their etiology, which are more limited, the clinical entities and the economic consideration in the decision to treat. Due to the latter, the number of antimycotic drugs available for the treatment of animals is significantly more restricted than those accessible in human medicine, especially for the treatment of disseminated infections. Even for drugs beyond their patent life, high prices limit their adoption in veterinary medicine [14].

This Special Issue on Therapy of Mycoses illuminates for the reader the state of the art in treatment considerations for major invasive and superficial human mycoses, as well as those in the arena of veterinary interest. As such, this issue is unique and of interest for a significant proportion of the scientific and clinical community interested in infectious diseases.
